# Saccade Amplitude and Pupil Diameter Information Channels: Extending the Gaze Information Channel Framework and Assessing Cross-Channel Association in Eye Tracking of Van Gogh Paintings

**DOI:** 10.3390/e28070767

**Published:** 2026-07-04

**Authors:** Marius Vila, Qiaohong Hao, Miquel Feixas, Micaela Y. Martin, Mateu Sbert

**Affiliations:** 1 Institute of Informatics and Applications, University of Girona, 17003 Girona, Spain; miquel.feixas@udg.edu; 2 College of Intelligent Engineering, Henan Institute of Technology, Xinxiang 453003, China; haoqiaohong@hait.edu.cn; 3 Institute of New Image Technologies, Universitat Jaume I, 12071 Castelló de la Plana, Spain; micmarti@uji.es

**Keywords:** gaze information channel, eye tracking, saccade amplitude, pupil diameter, Markov chain, Shannon entropy, mutual information, Van Gogh paintings, computational aesthetics

## Abstract

The gaze information channel paradigm models fixation sequences as a first-order Markov chain and quantifies gaze behaviour through Shannon entropy and mutual information (MI), where I(X;Y) measures the reduction in uncertainty about the next fixation state given the current one. This paper extends the framework by introducing two new channels: the saccade amplitude channel, which discretises saccade angular distance into three categories (short, medium, long) with a four-category variant also analysed, and the pupil diameter channel, which discretises fixation-period pupil size into three categories. Both are applied to 10 observers viewing 12 Van Gogh paintings. The amplitude channel shows that observer-driven variation exceeds stimulus-driven variation. The pupil channel yields the highest I(X;Y) among the two new channels (0.489±0.209 bits per participant), consistent with the slow dynamics of pupil responses. Goodness-of-fit tests confirm significantly non-random sequential structure in both channels (p<0.01) for all pooled matrices. A simultaneous cross-channel association analysis across all five channels finds that 19 of 20 pairwise Spearman correlations are non-significant; the single nominally significant result (pupil–duration, ρ=+0.697, p=0.025) does not survive Bonferroni correction and is not robust to outlier removal. Two theoretical observations are presented: an upper bound on conditional entropy in terms of transition persistence (Proposition 1), and a refinement monotonicity result showing that finer discretisation cannot decrease channel MI (Remark 2). An exploratory comparison with five computational aesthetics measures finds a nominally significant negative correlation between pupil I(X;Y) and Bense’s palette redundancy (ρ=−0.692, p=0.013, uncorrected), suggesting that diverse colour palettes are associated with stronger sequential pupil dynamics; permutation entropy and statistical complexity show no association with any channel.

## 1. Introduction

Eye tracking is one of the most direct windows into human visual cognition. The gaze information channel paradigm [1] models eye-tracking fixation sequences between Areas of Interest (AOIs) as a first-order Markov chain and derives a discrete information channel from the transition matrix. The stationary entropy H(X) quantifies how uniformly the observer distributes gaze across regions; the conditional entropy H(Y|X) quantifies the randomness of gaze transitions; and the mutual information I(X;Y)=H(X)−H(Y|X) measures sequential dependence.

A fixation-to-fixation trajectory carries information along several complementary dimensions simultaneously: which spatial region the gaze lands in (AOI), how long the fixation lasts (duration), how large the subsequent saccade is (amplitude), and in which direction the saccade travels (direction). Hao et al. introduced both the fixation duration channel [2] and the saccade direction channel [3].

This paper makes two methodological contributions: it introduces the saccade amplitude channel and the pupil diameter channel. These two channels were selected on complementary grounds. Saccade amplitude complements the previously introduced direction channel and represents the magnitude component of the saccadic displacement vector, constituting a natural next step in the same geometric decomposition. Pupil diameter, by contrast, is the only channel that is not purely spatial or temporal in nature: it is often associated with arousal and cognitive load [4] and therefore introduces a qualitatively new physiological dimension into the multi-channel framework, complementing the four purely oculomotor channels. Prior work on saccade amplitude has focused on its marginal distribution [5,6] or on high-order Markovian patterns of short and long saccade lengths (pixel displacement) without computing H(X), H(Y|X), or I(X;Y) [7], and prior work on pupil diameter has treated it as a scalar physiological signal [4] or used a temporally quantised marginal distribution [8], rather than modelling the sequence of fixation-period pupil states as a communication channel. With all five channels computed on the same dataset, we perform a simultaneous assessment of cross-channel association among their I(X;Y) summary statistics, using Spearman rank correlations computed both across participants and across paintings, with Bonferroni correction for multiple comparisons.

We also derive two theoretical observations (Proposition 1 and Remark 2): a simple upper bound on the conditional entropy in terms of the expected self-transition probability, and a refinement monotonicity result showing that state-space refinement cannot decrease channel mutual information.

The paper is organised as follows. Section 2 reviews related work. Section 3 describes the methodology. Section 4 presents results. Section 5 discusses the findings, Section 6 discusses limitations, and Section 7 concludes.

## 2. Related Work

*Notation note.* Throughout this paper the notation $H\left(X\right)\equiv H_{s}$ (stationary entropy) and $H\left(Y|X\right)\equiv H_{t}$ (transition entropy) are used interchangeably following the conventions established in the literature reviewed below; the former follows Cover and Thomas [9], the latter follows Krejtz et al. [10].

### 2.1. Gaze Entropy and Markov Chain Models

Ellis and Stark [11] were among the first to model fixation sequences as a Markov chain, computing conditional entropy from the transition matrix. Ponsoda et al. [12] constructed transition matrices over saccade direction categories. Krejtz et al. [10] formalised the stationary entropy $H_{s}$ and transition entropy $H_{t}$ as measures of attention dispersion and transition randomness. Shiferaw et al. [4] provide a comprehensive review of gaze entropy as a measure of visual scanning efficiency.

### 2.2. The Gaze Information Channel

Hao et al. [13] proposed extending the Markov chain model to a full information channel X→Y for Area of Interest (AOI) sequences, enabling computation of H(X,Y) and I(X;Y) in addition to $H_{s}$ and $H_{t}$. The AOI channel was developed fully by Hao et al. [1], who compared channel measures with computational aesthetics quantities and observed a visual correspondence between the curve of normalised AOI MI across Van Gogh’s chronological periods and the compressibility measure $M_{k}$ (defined in Section 2.4)—a relationship we revisit formally in Section 4.11. Hao et al. [13] applied the same AOI framework to scientific poster reading, demonstrating that MI correlates with cognitive comprehension.

### 2.3. Duration and Direction Channels

The fixation duration channel was introduced by Hao et al. [2], who classified fixations as short-duration (SD, <150 ms), medium-duration (MD, 150–900 ms), or long-duration (LD, ≥900 ms). They found that observer variation is more pronounced than image variation for the duration channel—in contrast to the pattern observed in the AOI channel.

**Figure 1:**
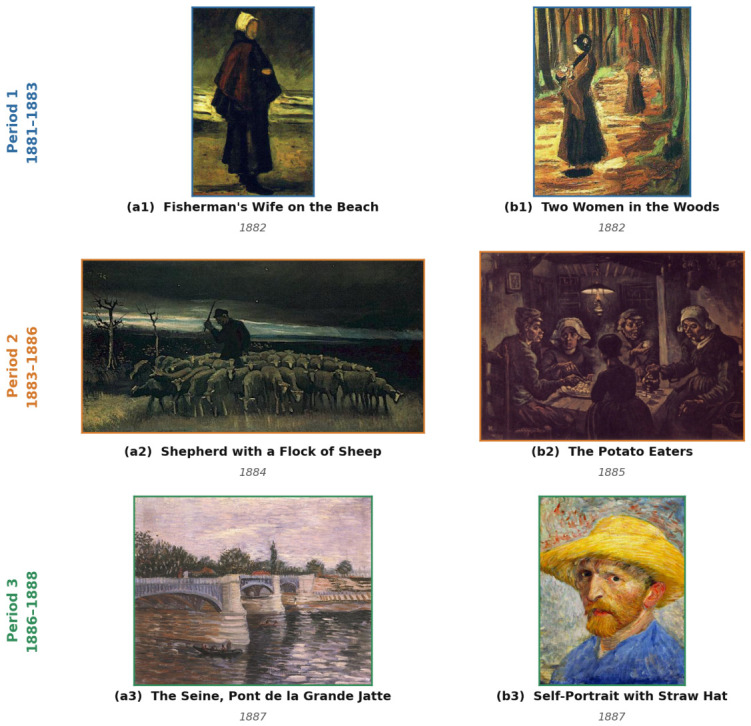

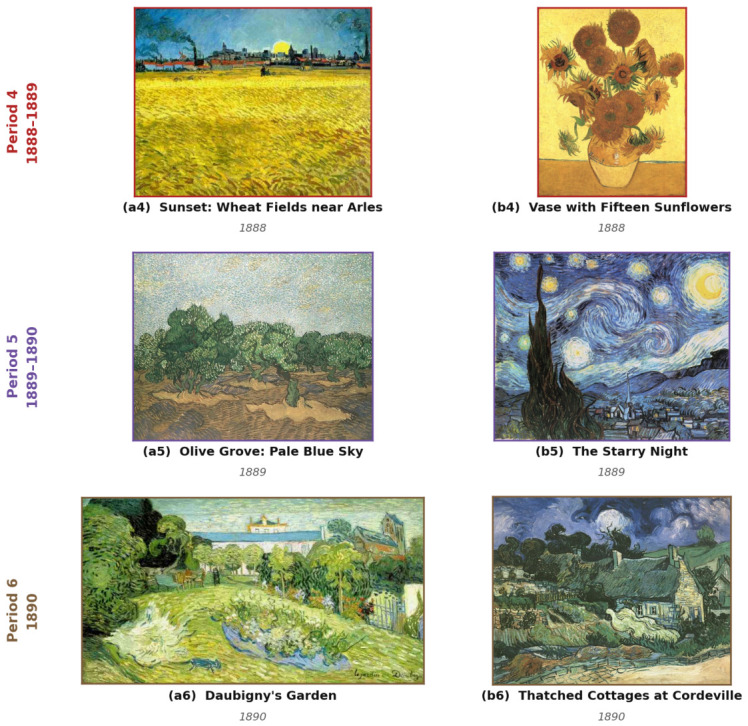
The 12 Van Gogh paintings used in the experiment: (**a1**) *Fisherman’s Wife on the Beach*; (**b1**) *Two Women in the Woods*; (**a2**) *Shepherd with a Flock of Sheep*; (**b2**) *The Potato Eaters*; (**a3**) *The Seine, Pont de la Grande Jatte*; (**b3**) *Self-Portrait with Straw Hat*; (**a4**) *Sunset: Wheat Fields near Arles*; (**b4**) *Vase with Fifteen Sunflowers*; (**a5**) *Olive Grove: Pale Blue Sky*; (**b5**) *The Starry Night*; (**a6**) *Daubigny’s Garden*; (**b6**) *Thatched Cottages at Cordeville*. The paintings arranged by chronological period (rows) and group a/b (columns). Credits (except (**a3**) and (**b2**)): The Vincent Van Gogh Gallery, David Brooks (http://www.vggallery.com (accessed on 9 March 2026), © 1996–2022). Credits for (**a3**) and (**b2**): Van Gogh Museum, Amsterdam (Vincent van Gogh Foundation).

The saccade direction channel was introduced by Hao et al. [3], using eight 45° directional bins. They found that the direction channel also discriminates better between observers than between paintings, and that mutual information is higher for horizontal/vertical saccades than for diagonal ones, with the exception of paintings with strong diagonal composition such as b5 (*The Starry Night*; see Figure 1).

These three published channels—AOI, fixation duration, and saccade direction—form the established background against which the present paper introduces the amplitude and pupil diameter channels.

To position these contributions, it is useful to compare three strands of prior work. Classical gaze entropy work (Ellis and Stark [11]; Krejtz et al. [10]; Shiferaw et al. [4]) characterises transition structure between spatial regions or directions using $H_{s}$ and $H_{t}$, but does not compute mutual information and does not treat the transition matrix as a full communication channel. Marginal amplitude studies (Foulsham and Kingstone [5]; Tatler and Vincent [6]; Bonev et al. [7]) have analysed the distribution and Markovian patterns of saccade lengths (pixel displacement) in relation to image content and task demands, but have not computed H(X), H(Y|X), or I(X;Y) for amplitude sequences. Prior pupillometry work (Mathôt [14]; Shiferaw et al. [4]) has analysed pupil diameter either as a scalar psychophysiological signal [15] or marginal distributions [8], without modelling the sequential transition structure between fixation-period pupil states as a Markov information channel. To the best of our knowledge, no prior work has modelled saccade amplitude or fixation-period pupil diameter sequences as a discrete information channel—constructing the transition matrix between consecutive fixation states, estimating the stationary distribution, and computing H(X), H(Y|X), and I(X;Y).

### 2.4. Computational Aesthetics and Van Gogh

Rigau et al. [16,17] proposed a suite of information-theoretic aesthetics measures grounded in Birkhoff’s order/complexity framework and applied them to Van Gogh’s chronological evolution. Three measures are relevant here. Bense’s palette redundancy $M_{B}=\left(H_{\max}-H\left(C\right)\right)/H_{\max}$ measures the reduction in uncertainty achieved by the palette relative to the maximum possible entropy, where H(C) is the Shannon entropy of the colour histogram and $H_{\max}=\log_{2}\left|C\right|$ is the maximum possible entropy for that colour space; high $M_{B}$ indicates a limited, homogeneous colour selection, while low $M_{B}$ indicates a heterogeneous, diverse palette. Kolmogorov compressibility $M_{k}=\left(NH_{\max}-K\left(I\right)\right)/\left(NH_{\max}\right)$ approximates the degree of order in the pixel arrangement via lossless compression, where *N* is the number of pixels and K(I) is the compressed file size in bits; higher $M_{k}$ means greater structural regularity. Compositional complexity $M_{s}^{-1}\left(0.25\right)$ is defined as the number of colour-homogeneous spatial regions required to account for 25% of the mutual information between the colour histogram and a spatial partition of the image; it quantifies how many distinct compositional elements a painting contains, with larger values indicating richer structural organisation. Across Van Gogh’s six chronological periods—Earliest Paintings (1881–1883), Nuenen/Antwerp (1883–1886), Paris (1886–1888), Arles (1888–1889), Saint-Rémy (1889–1890), and Auvers-sur-Oise (1890)—$M_{B}$ and $M_{k}$ decrease monotonically from Nuenen to Auvers (reflecting increasing colour diversity and structural complexity), while $M_{s}^{-1}$ increases (reflecting richer compositions) [17].

Sigaki et al. [18] computed two further pixel-level measures for nearly 140,000 paintings spanning a millennium of art history. The normalised permutation entropy $H_{\mathrm{PE}}$ is estimated from the ordinal probability distribution of 2×2 pixel patches: values near 1 indicate that local pixel arrangements are nearly random, while values near 0 indicate high local regularity. The statistical complexity $C_{\mathrm{PE}}=H_{\mathrm{PE}}\cdot Q_{\mathrm{JS}}$ (where $Q_{\mathrm{JS}}$ is the normalised Jensen–Shannon divergence between the ordinal pattern distribution and the uniform distribution) is maximised for processes that are neither fully ordered nor fully disordered, and equals zero at both extremes. The Van Gogh dataset was chosen because AOI-based channel measures have already been computed for it [1], enabling direct cross-channel comparison.

## 3. Method

### 3.1. Gaze Information Channel

Let the gaze sequence be represented as $X_{1},X_{2},\ldots ,X_{n}$, where each state belongs to a finite alphabet S={1,…,s}. The sequence is modelled as a first-order Markov chain with transition matrix $P=[p_{ij}]$, estimated by normalising transition counts row-wise. Under mild conditions the chain has a unique stationary distribution π satisfying πP=π.

Each signal dimension (amplitude, duration, direction, pupil) is modelled as an independent first-order Markov chain. This per-channel design follows the established gaze information channel framework [1,2,3] and is motivated by tractability: a joint model over all five channels would require a state space of size $O(s_{1}\times s_{2}\times \ldots \times s_{5})$, rendering transition-count estimation infeasible at the available sample sizes (N≈1000–3000 fixations per observer). Inter-channel statistical dependencies are instead examined explicitly through the cross-channel Spearman correlation analysis in Section 4.10. This two-stage approach—independent characterisation followed by pairwise association testing—is consistent with prior work on sequential eye-movement analysis [11,12,19].

### 3.2. Channel Measures

Following [1], with the convention 0log0=0:

(1) $$H_{s}=H\left(X\right)=H\left(Y\right)=-\sum_{i}\pi_{i}\log_{2}\pi_{i}$$

(2) $$H_{t}=H\left(Y|X\right)=-\sum_{i}\sum_{j}\pi_{i}\;p_{ij}\log_{2}p_{ij}$$

(3) H(X,Y)=H(X)+H(Y|X)

(4) I(X;Y)=H(X)−H(Y|X)

The normalised mutual information I(X;Y)/H(X) is scale-free, allowing comparison across channels with different state-space sizes. All logarithms are base 2 (bits). Unless otherwise stated, amplitude channel results refer to the 3-category scheme (Short/Medium/Long), and values are pooled across participants (for per-painting results) or across paintings (for per-participant results).

We derive a simple upper bound on the conditional entropy in terms of the expected self-transition probability. While the ingredients are standard, this formulation provides a direct link between transition persistence and mutual information in gaze channels.

**Proposition 1** 
(Persistence and conditional entropy)**.** *Let $\left(X_{t}\right)$ be a stationary first-order Markov chain on a finite state space S with |S|=m, stationary distribution π, and transition matrix $P=[p_{ij}]$. Define the expected self-transition probability*

$$\alpha =\sum_{i\in S}\pi_{i}\;p_{ii}.$$

*Then, we denote by $H_{b}\left(p\right)=-p\log_{2}p-\left(1-p\right)\log_{2}\left(1-p\right)$ the binary entropy function,*

$$H\left(Y|X\right)\leq H_{b}\left(\alpha \right)+\left(1-\alpha \right)\log_{2}\left(m-1\right).$$

*That is, the conditional entropy of the channel output is bounded above by a function of persistence α and state-space size m alone, regardless of the off-diagonal transition structure.*

**Proof.**  For each state *i*, among all distributions over *m* states with fixed self-probability $p_{ii}$, the conditional entropy H(Y|X=i) is maximised when the remaining probability mass $1-p_{ii}$ is spread uniformly over the other m−1 states [9]. Hence

$$H\left(Y|X=i\right)\leq H_{b}\left(p_{ii}\right)+\left(1-p_{ii}\right)\log_{2}\left(m-1\right).$$

Averaging with respect to π gives

$$H\left(Y|X\right)\leq \sum_{i}\pi_{i}H_{b}\left(p_{ii}\right)+\left(1-\alpha \right)\log_{2}\left(m-1\right).$$

Since $H_{b}$ is concave, Jensen’s inequality yields $\sum_{i}\pi_{i}H_{b}\left(p_{ii}\right)\leq H_{b}\left(\alpha \right)$, so $H\left(Y|X\right)\leq H_{b}\left(\alpha \right)+\left(1-\alpha \right)\log_{2}\left(m-1\right)$. □

**Remark 1.** 
*The proposition shows that the conditional entropy H(Y|X) of a gaze channel is controlled by two factors: the persistence α (which sets the upper bound) and the off-diagonal transition structure (which determines how far below the bound the actual H(Y|X) falls). Since I(X;Y)=H(X)−H(Y|X), a lower upper bound on H(Y|X) increases the maximum attainable mutual information for a fixed H(X): channels with strong persistence can achieve higher MI, though they are not guaranteed to. The empirical tightness of this bound—how much slack remains between the bound and the actual H(Y|X) for each channel—is examined in Section 5.*

**Remark 2.** 
*Since the 3-category amplitude scheme is obtained from the 4-category scheme by deterministic merging of states, the data-processing inequality [9] implies*

$$I_{3-\mathrm{cat}}\left(X;Y\right)\leq I_{4-\mathrm{cat}}\left(X;Y\right).$$

*Therefore the increase in MI observed in Table 1 (participants 0.029→0.040 bits; paintings 0.026→0.037 bits) is expected theoretically whenever one categorisation is a refinement of the other.*

**Practical interpretation.** Proposition 1 connects the persistence parameter α to an upper bound on H(Y|X) and hence to I(X;Y). A high α (strongly persistent gaze, e.g., long fixations within a single AOI or slow, large-amplitude saccades) implies a tight bound on conditional entropy, predicting high sequential predictability and high MI. This allows researchers to rank channels or observers by their degree of gaze “stickiness” using a single observable statistic (α) before computing the full channel; the slack values reported in Section 5 confirm that the bound is informative rather than vacuous. Remark 2 provides a principled basis for calibrating discretisation: since adding categories can only increase MI, a researcher can systematically refine the binning and monitor diminishing returns. For the amplitude channel, moving from 3 to 4 categories increases mean participant MI by 0.011 bits (0.029→0.040 bits)—a small gain that confirms the 3-category scheme already captures most of the sequential structure and is adequate for cross-channel comparisons.

### 3.3. Categorisation Schemes and Validation

**Saccade amplitude channel.** The visual angle θ of each saccade is computed from pixel displacement *d* using a screen-to-eye distance of 700 mm and 0.2745 mm/px:

(5) $$\theta =2\cdot \arctan\;\left(\frac{d\times 0.2745}{2\times 700}\right)\times \frac{180}{\pi }\;\left(\mathrm{degrees}\right)$$

The *3-category scheme* partitions saccades into short ($\theta <1^{\circ }$), medium ($1^{\circ }\leq \theta \leq 5^{\circ }$), and long ($\theta >5^{\circ }$). The *4-category scheme* further splits at $3^{\circ }$: very-short ($\theta <1^{\circ }$), short ($1^{\circ }$–$3^{\circ }$), medium ($3^{\circ }$–$5^{\circ }$), long ($>5^{\circ }$). The 1° and 5° boundaries follow established functional distinctions in the scene-viewing literature: saccades below 1° are typically classified as microsaccades or very short corrective movements; the 1°–5° range encompasses the ambient-to-focal transition zone typical of art and scene viewing [5,6,20]; and saccades exceeding 5° constitute large exploratory re-orientations. The 3° split in the 4-category scheme further separates within-region (1°–3°) from between-region (3°–5°) ambient saccades. The robustness of the choice is confirmed empirically by Remark 2: the 3-category and 4-category schemes yield nearly identical normalised MI (see Section 4.6), demonstrating that the key information content is captured by the 3-category scheme and that the results are not sensitive to the precise location of the boundary within the medium range. In 18 of the 120 participant–painting pairs (15%), the long category is empty; in these cases measures are computed over non-empty categories only.

**Figure 2:**
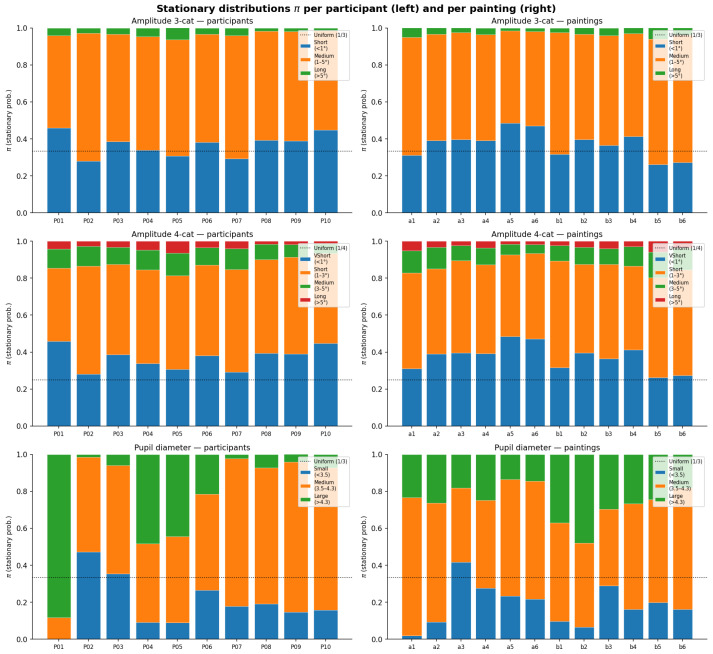
Stationary distributions π per participant (**left**) and per painting (**right**). Top row: amplitude 3-category scheme; middle row: amplitude 4-category scheme; bottom row: pupil diameter channel. For the 3-category amplitude channel, medium saccades (1–5°) dominate ($\pi_{\mathrm{medium}}\approx 0.58$); long saccades are rare ($\pi_{\mathrm{long}}\approx 0.06$). For the 4-category scheme, the medium band is split at 3°: short (1°–3°) and medium (3°–5°) saccades together account for the same dominant mass. For the pupil channel, medium-diameter fixations dominate the stationary distribution ($\pi_{\mathrm{medium}}\approx 0.59$), with large fixations accounting for the remainder ($\pi_{\mathrm{large}}\approx 0.31$) and small fixations rare ($\pi_{\mathrm{small}}\approx 0.10$). Dotted line: uniform distribution ($\pi_{i}=1/n$ for *n* states).

**Pupil diameter channel.** Mean pupil diameter during each fixation period is classified as small (S, <3.5 mm), medium (M, 3.5–4.3 mm), or large (L, >4.3 mm). Pupil size is influenced by cognitive arousal and mental effort via the psychosensory pupil response [14], in addition to luminance-driven mechanisms. During free viewing of paintings, pupil dilation has been shown to track high-level perceptual processing: observers show larger pupils when judging the quality of abstract art, suggesting that pupil size indexes cognitive engagement with the stimulus [15]. These thresholds were chosen to separate qualitatively distinct pupil-diameter ranges (small, medium, and large); the observed empirical proportions (Small ≈9.9%, Medium ≈32.7%, Large ≈57.4%) reflect the raw frequency of each category across all fixations, while the stationary distribution π derived from the transition matrix gives a somewhat different picture (see Figure 2, bottom row). A total of 132 fixations with diameter ≤0.5 mm were excluded as tracking artefacts (0.95% of 13,897 total fixations); these affect only the transition matrix construction. Unlike the amplitude channel, we do not introduce a 4-category pupil scheme. One methodological limitation of the pupil channel in painting-viewing studies is the luminance confound: as the eye moves between bright and dark regions of a painting, the pupil light response modulates diameter independently of cognitive arousal [14]. We do not apply luminance correction in this study because luminance values per fixation are not available in the dataset; the sequential structure captured by the channel therefore reflects a mixture of psychosensory and luminance-driven dynamics. A luminance-corrected replication is noted as future work. For amplitude, the split at $3^{\circ }$ within the medium band has a clear physiological motivation, refining the ambient/focal distinction. For pupil diameter, no equivalent physiological boundary motivates a fourth category; the three bins reflect the empirical distribution of pupil sizes, and finer bins would produce smaller counts in the extreme categories where data are already sparse.

**Validation.** To confirm that the observed sequential structure is non-random, we apply the Besag and Mondal [21] exact goodness-of-fit test for Markov chains to the pooled transition matrices (all 12 paintings per participant; all 10 participants per painting). The null hypothesis is $p_{ij}=\pi_{j}$ for all *i* (independence). For both new channels, all 10 matrices averaged across participants and all 12 matrices averaged across paintings reject $H_{0}$ at p<0.01, confirming that the sequential structure is significantly non-random.

### 3.4. Statistical Analysis

Distributional properties of I(X;Y) were assessed using the Shapiro–Wilk test applied to all N=120 individual observations (10 participants × 12 paintings). $I_{\mathrm{Dir}}$ was the only channel consistent with approximate normality (Shapiro–Wilk W=0.984, *p* = 0.174), where $I_{\mathrm{Ch}}$ denotes I(X;Y) for channel Ch; all remaining channels departed significantly (p<0.001 in each case, with *W* ranging from 0.672 for $I_{\mathrm{Amp}}$ to 0.927 for $I_{\mathrm{Pup}}$). All subsequent inferential analyses therefore use nonparametric procedures. Differences in I(X;Y) across the five channels were assessed with the Friedman test applied to participant-level data (N=10). Associations among I(X;Y) values and between I(X;Y) and image-complexity measures were examined with Spearman’s ρ. All tests are two-tailed with a nominal significance level of 0.05.

## 4. Results

### 4.1. Dataset

The dataset consists of eye-tracking recordings from 10 observers (five female, mean age 24.3 ± 3.1 years) viewing 12 Van Gogh paintings under free-viewing conditions (45 s per painting, SMI iViewETG2w eye tracker). Observers were recruited from the student and staff community of Tianjin University through posted notices; all had normal or corrected-to-normal vision and reported no history of colour deficiency. Ethical approval and informed consent procedures for the original data collection are described in [1]; the present study performs a secondary analysis of these previously collected, de-identified recordings and did not involve any new data collection. Observers were instructed to view each painting freely for 45 seconds, with no prescribed task, no mention of subsequent testing, and no instruction to memorise or evaluate the works. This free-viewing paradigm is standard in aesthetic eye-tracking research and stands in contrast to task-directed viewing, in which instructions are known to profoundly redirect gaze [22,23]. The 12 paintings span six chronological periods—Earliest Paintings (1881–1883), Nuenen/Antwerp (1883–1886), Paris (1886–1888), Arles (1888–1889), Saint-Rémy (1889–1890), and Auvers-sur-Oise (1890)—with two representative paintings selected per period. Following [1], the two paintings within each period are labelled group a and group b (an arbitrary distinction inherited from that work); see Figure 1. Full experimental details are given in [1].

### 4.2. Participant-Level Amplitude Analysis

**Figure 3:**
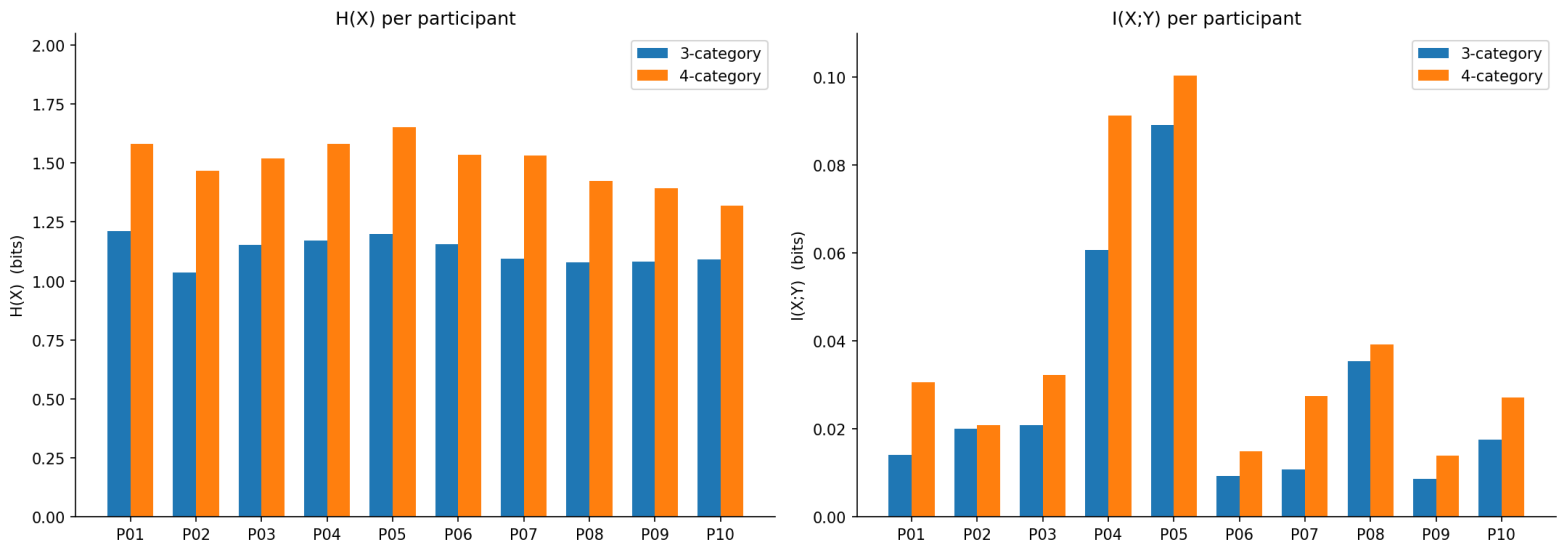
H(X) (**left**) and I(X;Y) (**right**) per participant. Blue bars: 3-category scheme; orange bars: 4-category scheme.

Table 1 (left panel) shows H(X) and I(X;Y) per participant for both amplitude schemes (Figure 3). H(X) is stable across participants (3-cat: mean 1.127, SD 0.059 bits), suggesting a broadly similar marginal distribution of saccade amplitudes across observers, although equal entropy values do not uniquely imply identical distributions. I(X;Y) shows more variability (3-cat: mean 0.029, SD 0.026 bits). The stationary distributions π for both amplitude schemes are shown in Figure 2 (top and middle rows): medium saccades ($1^{\circ }$–$5^{\circ }$) dominate the 3-category amplitude channel ($\pi_{\mathrm{medium}}\approx 0.58$), while the 4-category scheme reveals that this mass splits roughly equally between the short ($1^{\circ }$–$3^{\circ }$) and medium ($3^{\circ }$–$5^{\circ }$) bands.

### 4.3. Stimulus-Level Amplitude Analysis

**Figure 4:**
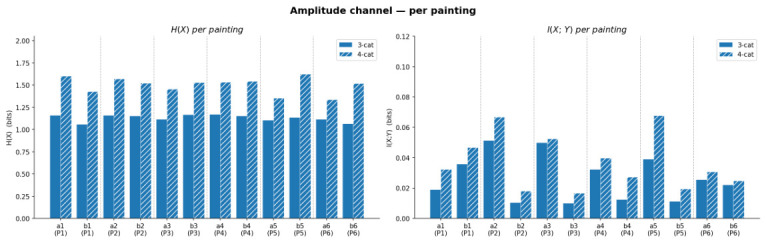
H(X) (**left**) and I(X;Y) (**right**) per painting for the 3-category (solid blue) and 4-category (hatched blue) amplitude schemes. Dashed vertical lines separate chronological periods.

Table 1 (right panel) shows channel measures per painting for both amplitude schemes (Figure 4). Inter-painting variation in I(X;Y) (3-cat: mean 0.026, SD 0.015 bits) is lower than inter-participant variation (3-cat: mean 0.029, SD 0.026 bits), suggesting that, in this dataset, observer-driven variation exceeds stimulus-driven variation for the amplitude channel.

### 4.4. MI by Chronological Period

**Figure 5:**
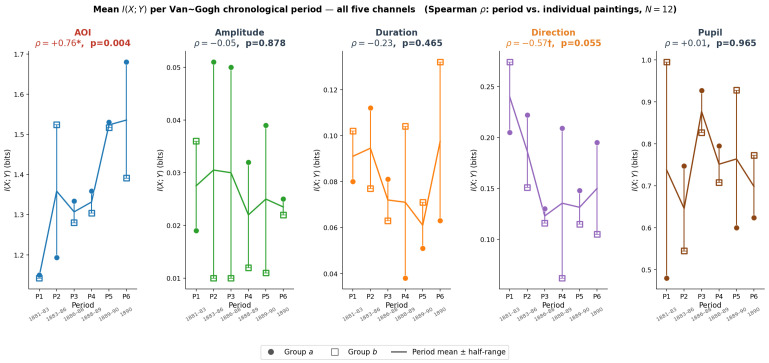
Mean I(X;Y) per Van Gogh chronological period for all five channels. Filled circles: group a paintings; open squares: group b paintings; solid line: period mean; error bars: ±half-range across the two paintings per period. Spearman ρ and *p*-value are computed on all 12 individual paintings (N=12); asterisk: p<0.05; dagger: p<0.10 (uncorrected). Only the AOI channel shows a statistically significant positive trend with period; the direction channel shows a marginal negative trend (ρ=−0.57, p=0.055); all other channels are flat.

Figure 5 shows the mean I(X;Y) per Van Gogh chronological period for all five channels. The AOI channel shows a significant positive trend (ρ=+0.763, p=0.004; see [1]), reflecting the growing compositional complexity of Van Gogh’s later work. In contrast, amplitude MI shows no monotonic trend (ρ=−0.050, p=0.878), confirming that while amplitude MI discriminates observers slightly better than paintings overall (results derived from Table 2 and summarized in Table 3, ratio 0.58), it is insensitive to the chronological evolution of Van Gogh’s style. Duration (ρ=−0.234, p=0.465) and pupil (ρ=+0.014, p=0.965) channels show no significant chronological trend. The direction channel shows a marginal negative trend (ρ=−0.565, p=0.055), suggesting that later Van Gogh periods may elicit slightly less predictable saccade direction sequences, though this does not survive correction for multiple comparisons. Overall, the AOI channel remains the only one to show a statistically significant association with Van Gogh’s stylistic evolution.

### 4.5. Five-Channel Summary

**Figure 6:**
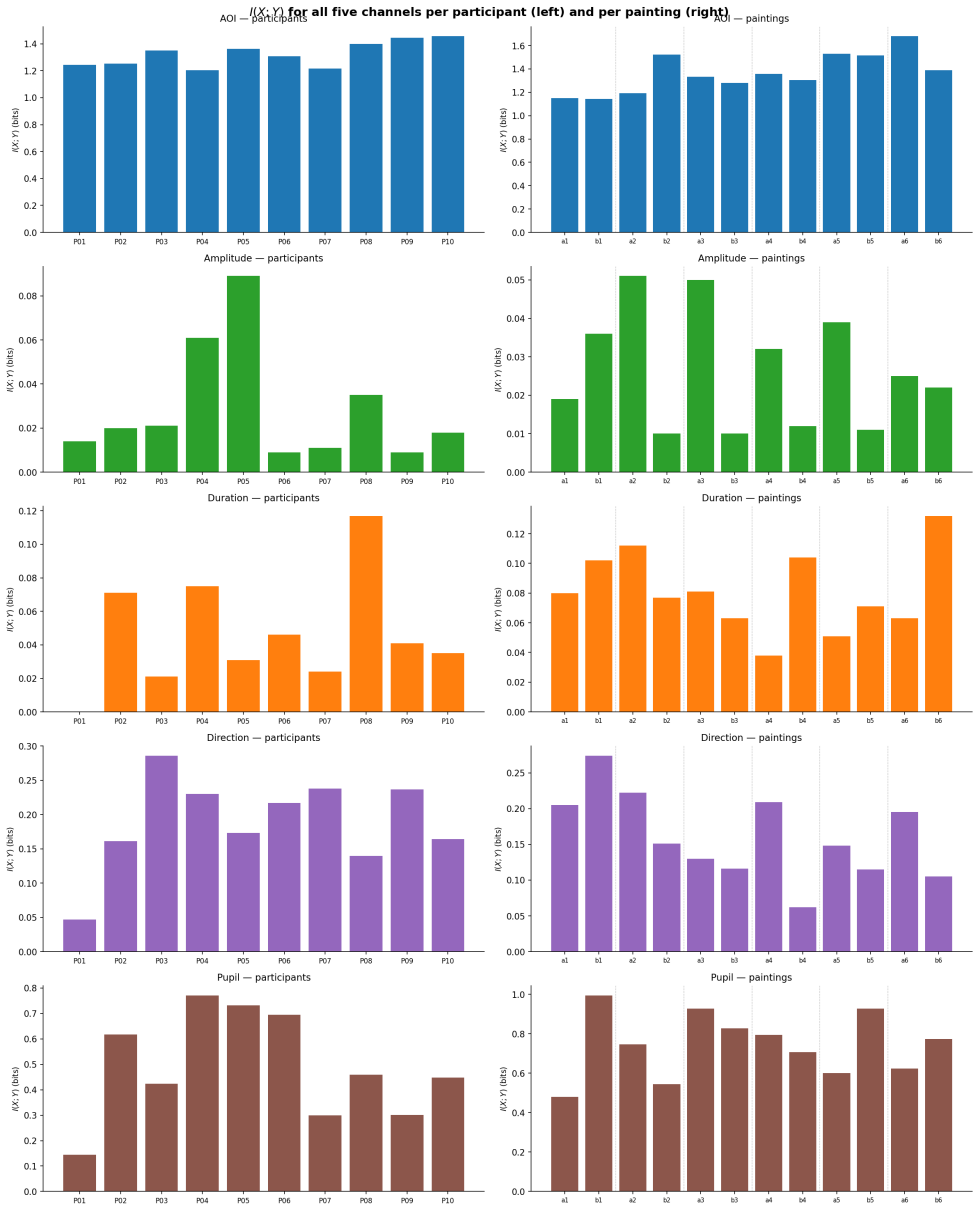
I(X;Y) for all five channels per participant (**left**) and per painting (**right**). Each channel has its own colour: AOI (blue), amplitude (green), duration (orange), direction (purple), pupil (brown). Dashed vertical lines separate chronological periods (paintings panel).

Table 2 (left panel: participants; right panel: paintings) summarises I(X;Y) from all five channels simultaneously. The most immediately striking feature is the scale separation between channels: AOI I(X;Y) (participant mean 1.324 bits; painting mean 1.367 bits) substantially exceeds all other channels, reflecting the large state space and rich spatial structure of the AOI representation. The pupil channel is the second highest contributor (participant mean 0.489 bits; painting mean 0.746 bits), attributable to the slow physiological dynamics of pupil dilation discussed in Section 5. The amplitude channel has the smallest I(X;Y) values (participant mean 0.029 bits; painting mean 0.026 bits), followed by the duration channel (participant mean 0.046 bits) and the direction channel (participant mean 0.189 bits). A Friedman test on participant-level data (N=10) confirmed that the five channels differ significantly in I(X;Y) ($\chi^{2}\left(4\right)=38.69$, p<0.001), with mean ranks ${\bar{r}}_{\mathrm{AOI}}=5.00>{\bar{r}}_{\mathrm{Pup}}=4.00>{\bar{r}}_{\mathrm{Dir}}=3.00>{\bar{r}}_{\mathrm{Dur}}=1.75>{\bar{r}}_{\mathrm{Amp}}=1.25$, confirming the scale separation visible in Table 2 and Figure 6. Within the participant panel, P01 is a notable outlier: it records zero MI for the duration channel and the lowest MI for the pupil channel (0.144 bits), suggesting an atypically uniform fixation-period pupil-diameter transition pattern. Within the painting panel, b1 (*Two Women in the Woods*) elicits the highest pupil MI (0.995 bits), while the amplitude channel is comparatively stable across all twelve paintings (range 0.010–0.051 bits). Table 3 shows that inter-participant variation in amplitude MI ($\sigma_{\mathrm{obs}}=0.026$ bits) exceeds inter-painting variation ($\sigma_{\mathrm{stim}}=0.015$ bits, ratio 0.58), indicating that amplitude sequential structure is more observer-specific than stimulus-driven—in contrast to the AOI channel (ratio 1.82).

Table 3 summarises the discrimination profile of each channel. Only the AOI channel (ratio 1.82) shows greater inter-painting variability than inter-participant variability, indicating that its sequential structure is tightly linked to stimulus content. All remaining channels—amplitude (0.58), duration (0.79), direction (0.90), and pupil (0.77)—show higher inter-participant variability, suggesting that observer-specific factors play the dominant role. The contrast between the AOI ratio (1.82) and the amplitude ratio (0.58) is noteworthy: although both channels classify saccade-related events, the spatial destination of a saccade is more strongly constrained by compositional features of the painting, while the distance travelled is relatively more observer-specific. Figure 6 visualises these patterns simultaneously across all five channels.

### 4.6. Normalised Mutual Information

**Figure 7:**
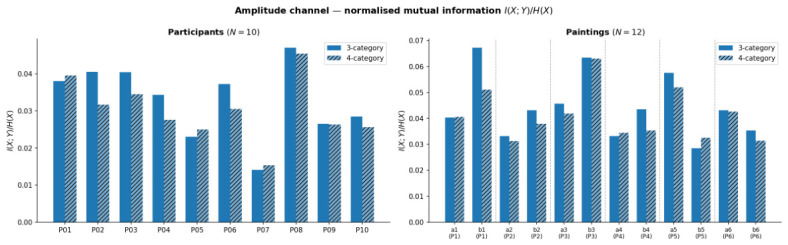
Normalised MI I(X;Y)/H(X) for the amplitude channel, per participant (**left**) and per painting (**right**). Blue: 3-category scheme; blue hatched: 4-category scheme. Values are nearly identical across the two schemes, confirming robustness to the choice of categorisation boundary (Remark 2).

Figure 7 shows the normalised MI I(X;Y)/H(X). Values are nearly identical across the 3-category and 4-category schemes, confirming robustness to the choice of categorisation boundary.

### 4.7. Transition Matrices

**Figure 8:**
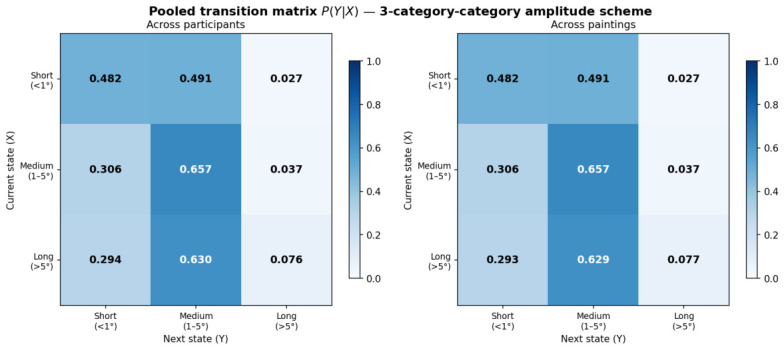
Pooled P(Y|X) for the 3-category amplitude scheme: across participants (**left**) and across paintings (**right**).

**Figure 9:**
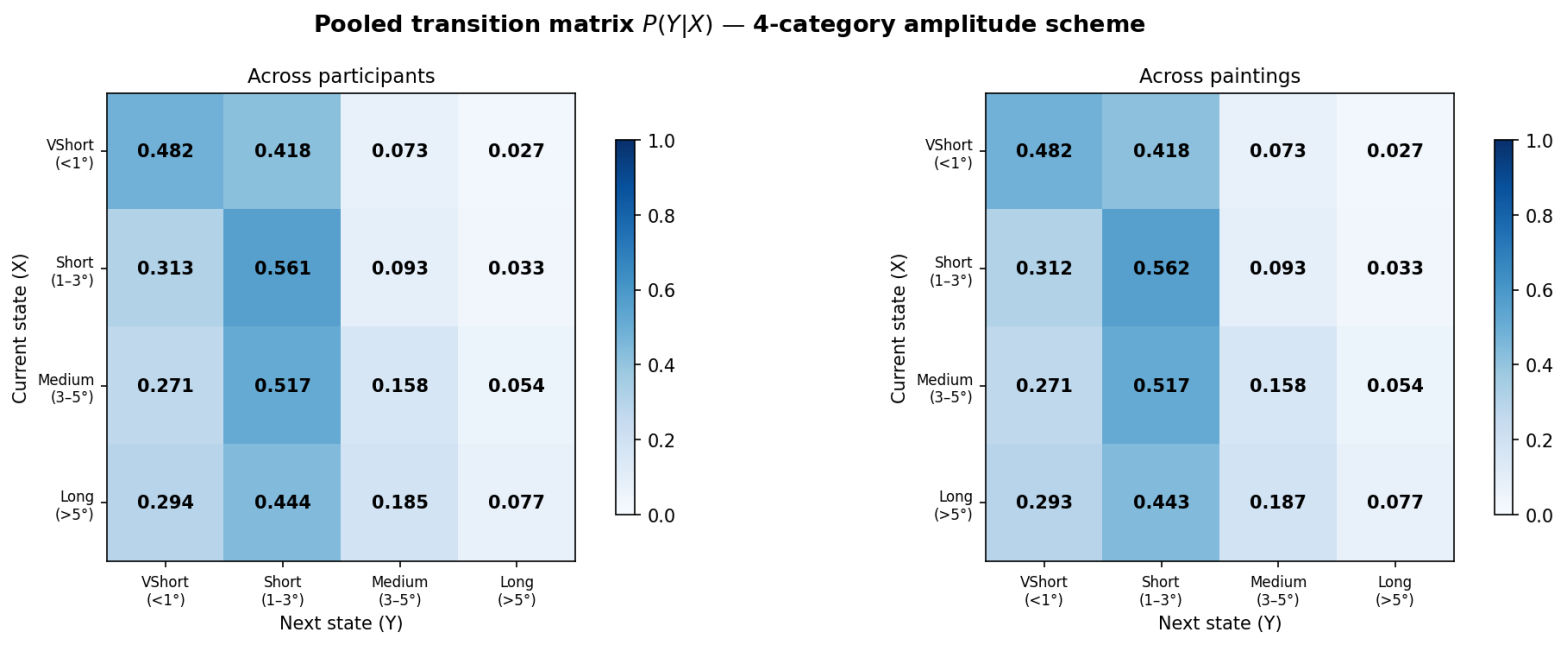
Pooled P(Y|X) for the 4-category amplitude scheme: across participants (**left**) and across paintings (**right**).

Figure 8 and Figure 9 show the pooled transition matrices P(Y|X). In the 3-category scheme (Figure 8), medium–medium self-transitions are the strongest ($p_{MM}\approx 0.657$), reflecting the dominance of medium-amplitude saccades in the stationary distribution. Long saccades have the lowest self-transition probability ($p_{LL}\approx 0.077$), as they are more likely to be followed by a short or medium saccade. In the 4-category scheme (Figure 9), very-short–very-short self-transitions tie with short–short at the highest level ($p_{VS}\approx p_{SS}\approx 0.48$–0.56), while medium–medium self-transitions are the weakest ($p_{MM}\approx 0.158$).

### 4.8. Participant-Level Pupil Analysis

**Figure 10:**
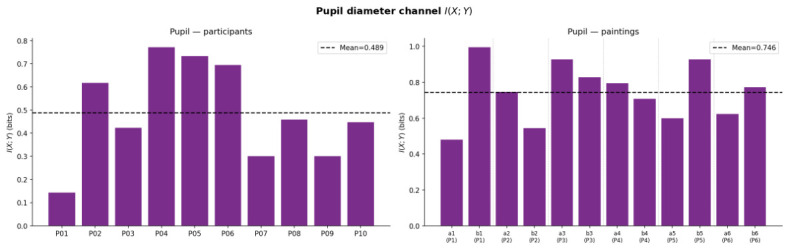
I(X;Y) of the pupil diameter channel per participant (**left**) and per painting (**right**). Dashed line: group mean. Dashed vertical lines separate chronological periods (paintings panel).

Table 4 and Figure 10 (left) show I(X;Y) per participant. The range is 0.144 (P01) to 0.771 (P04), with mean 0.489±0.209 bits. The stationary distribution π of the pupil channel is shown in Figure 2 (bottom row): the Medium state dominates ($\pi_{\mathrm{medium}}\approx 0.59$), with large fixations accounting for approximately 0.31 and small fixations rare (≈0.10). These values substantially exceed the amplitude and duration channels, and the pupil channel ranks second overall after the AOI channel.

Inspecting the per-painting panel, group b paintings tend to elicit higher pupil MI than their group a counterparts within the same period (means 0.796 vs. 0.696 bits), though with only six period pairs this pattern should be treated as descriptive.

### 4.9. Pupil Transition Matrices

**Figure 11:**
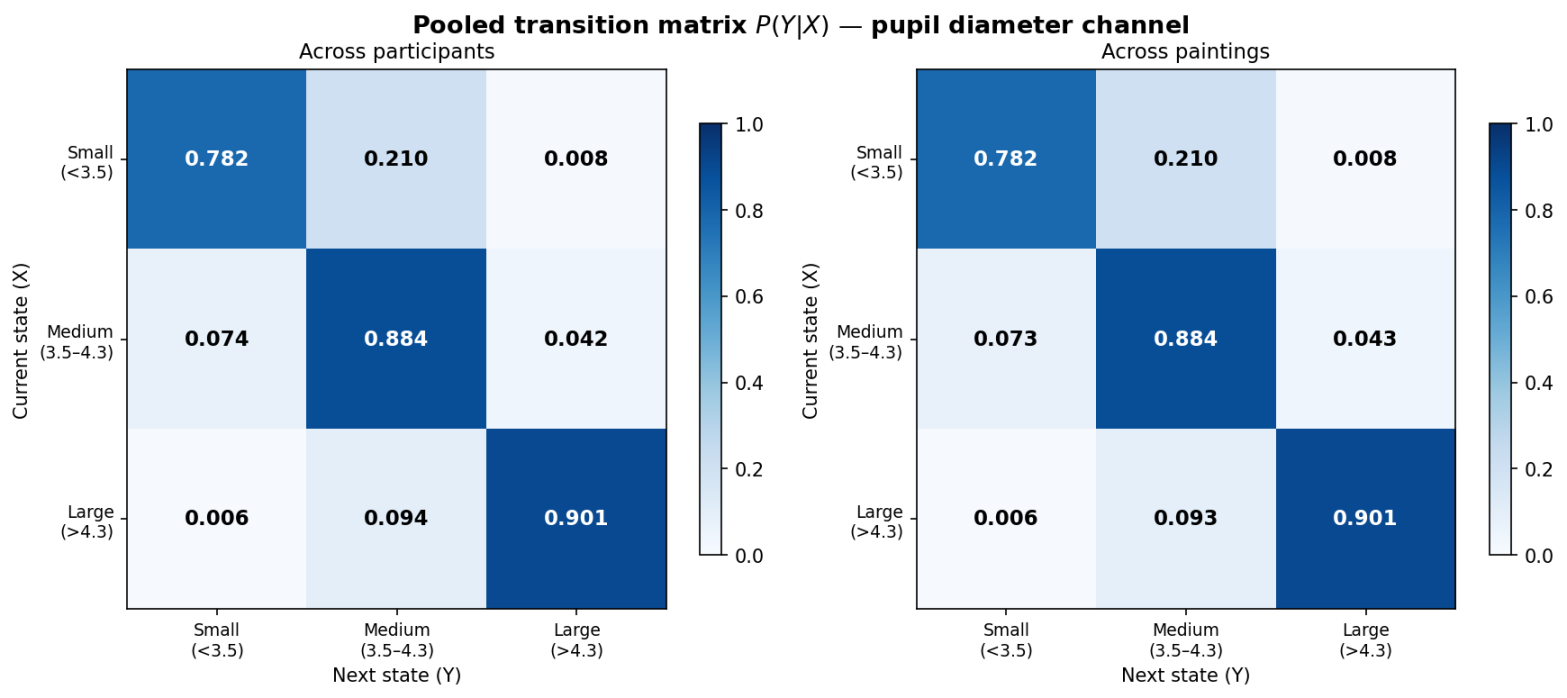
Pooled P(Y|X) for the pupil diameter channel: across participants (**left**) and across paintings (**right**).

Figure 11 shows P(Y|X) for the pupil channel, averaged separately across participants (left) and across paintings (right). Self-transition probabilities are very high in both cases, consistent with slow physiological persistence of pupil dilation. The two pooled matrices are nearly identical, with self-transition probabilities around 0.78 (Small), 0.88 (Medium), and 0.90 (Large), suggesting that pupil persistence is somewhat more consistent across paintings than across individuals—in line with the finding from Table 3 that the pupil channel discriminates observers slightly better than paintings. However, pupil diameter is also influenced by luminance, baseline differences across participants, and measurement conditions; these factors may contribute to the observed persistence independently of arousal.

### 4.10. Cross-Channel Association

Table 5 (left and right panels) presents the full 5 × 5 Spearman rank-correlation matrix between I(X;Y) values from all five channels. Given the small sample sizes (N=10 participants, N=12 paintings), these results should be interpreted as exploratory and require validation on larger datasets. Of the 20 off-diagonal entries, 19 did not reach statistical significance at a nominal significance level of 0.05 (uncorrected). Under Bonferroni correction within each correlation matrix for 10 unique channel pairs (threshold p<0.005), the single nominally significant result—Pupil–Duration (ρ=+0.697, p=0.025)—does not remain significant. Applying a correction across all 20 participant- and painting-level tests would be even more conservative and leads to the same conclusion.

A plausible interpretation is that both the pupil and duration channels are sensitive to observer-level differences in cognitive engagement or arousal. At the painting level, the correlation did not reach statistical significance (ρ=+0.028, p=0.931).

**Figure 12:**
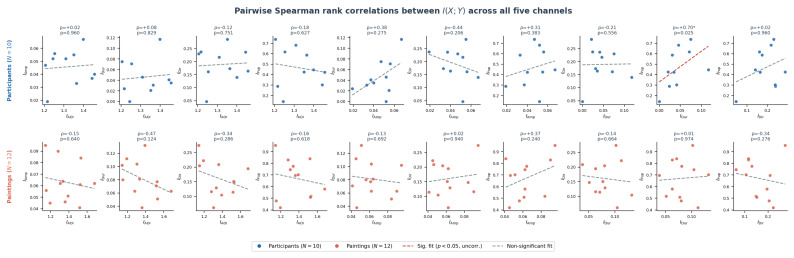
Pairwise Spearman rank correlations between I(X;Y) across all five channels. Each panel shows the scatter of I(X;Y) values for one channel pair, with the Spearman ρ and two-tailed *p*-value annotated at the top (asterisk: p<0.05, uncorrected). (**Top row**): participant-level data (N=10, blue circles); (**bottom row**): painting-level data (N=12, orange circles). Red dashed line: linear fit for the nominally significant pair (Duration–Pupil, participants: ρ=+0.70, p=0.025); grey dashed lines: non-significant fits. The Duration–Pupil result does not survive Bonferroni correction and is not robust to removal of one outlier participant (Section 4.10).

**Sensitivity analysis.** The baseline per-participant I(X;Y) values used in the cross-channel analysis are given in Table 2 (left panel). Inspection of the scatter plots (Figure 12) reveals that P01 is an outlier across several channel combinations: it has the lowest I(X;Y) in both the pupil channel (0.144, z=−1.79) and the direction channel (0.047, z=−2.22), and zero MI in the duration channel. The baseline Duration–Pupil correlation with the full sample (N=10) is ρ=+0.697, p=0.025. When P01 is removed (N=9), this correlation loses significance (ρ=+0.583, p=0.099), indicating that the Duration–Pupil association is substantially driven by a single participant and is not robust.

### 4.11. Relationship with Computational Aesthetics Measures (Exploratory Analysis)

Hao et al. [1] compared AOI channel measures with three computational aesthetics quantities computed from the pixel structure of the same 12 paintings: Kolmogorov complexity-based compressibility $M_{k}$, Bense’s palette redundancy $M_{B}$ [16], and compositional complexity $M_{s}^{-1}$ [16]. They found that the curve of normalised AOI I(X;Y) across paintings closely follows $M_{k}$ and $1-M_{B}$. With all five channels now available on the same dataset, we can extend this comparison. Per-painting values of $M_{k}$, $M_{B}$, and $M_{s}^{-1}\left(0.25\right)$ for all 12 stimuli were extracted from [1,17]. In addition, we compute the normalised permutation entropy $H_{\mathrm{PE}}$ and statistical complexity $C_{\mathrm{PE}}$ for each painting following Sigaki et al. [18], using a 2×2 sliding window (24 ordinal patterns) applied to the greyscale image. Full definitions of all five measures are given in Section 2.4.

Table 6 shows the Spearman rank correlations between each channel’s I(X;Y) per painting and all five aesthetics measures.

All results should be treated with caution given the small number of paintings (N=12) and the multiple comparisons involved (25 pairs tested across five measures).

The strongest association is between the pupil channel and Bense’s palette redundancy (ρ=−0.692, p=0.013, uncorrected): paintings with a more heterogeneous, colouristically diverse palette are associated with stronger sequential pupil dynamics. One possible interpretation is that a richer palette engages the psychosensory pupil response more continuously across fixations, although luminance-driven contributions cannot be excluded (see Section 6). The direction channel shows a marginal positive correlation with $M_{k}$ (ρ=+0.510, p=0.090) and the AOI channel with compositional complexity $M_{s}^{-1}$ (ρ=+0.503, p=0.095); the amplitude and duration channels show no significant association with any image-based measure. The $H_{\mathrm{PE}}$ and $C_{\mathrm{PE}}$ columns show no significant associations with any channel (all |ρ|≤0.48, all p≥0.117), likely because all 12 Van Gogh paintings occupy a uniformly high entropy range (Table 7: 0.934–0.995) that limits between-painting discrimination.

## 5. Discussion

The robustness of the amplitude channel across two categorisation schemes confirms that the sequential structure it captures is not an artefact of the particular bin boundaries chosen, a property guaranteed theoretically by Remark 2 and verified empirically by the near-identical normalised MI values (Section 4.6). The dominance of medium–medium self-transitions is consistent with the ambient/focal framework of visual attention [10], suggesting that observers tend to maintain their saccade amplitude regime across consecutive fixations rather than alternating freely between scales.

**Figure 13:**
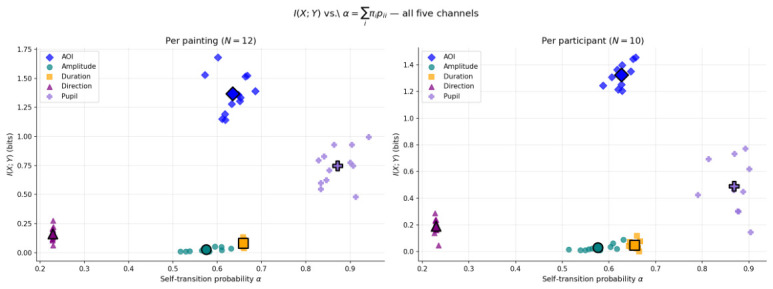
Scatter plot of I(X;Y) (vertical axis) vs. expected self-transition probability $\alpha =\sum_{i}\pi_{i}p_{ii}$ (horizontal axis) for all five channels: AOI (diamonds), amplitude (circles), duration (squares), direction (triangles), pupil (crosses). (**Left**): per painting (N=12); (**right**): per participant (N=10). Large filled markers with black outlines indicate the channel grand mean. The direction and amplitude channels have the lowest α and low–moderate MI; AOI and duration have intermediate α; the pupil channel has the highest α and the highest MI among the four non-AOI channels. AOI is the notable exception: moderate α but by far the highest I(X;Y), reflecting that its large state space and rich spatial structure dominate its mutual information beyond the persistence effect.

The link between transition persistence and mutual information, formalised in Proposition 1, is illustrated across all five channels in Figure 13, which plots I(X;Y) (vertical axis) vs. the expected self-transition probability $\alpha =\sum_{i}\pi_{i}p_{ii}$ (horizontal axis).

We verified the Proposition 1 bound $H\left(Y|X\right)\leq H_{b}\left(\alpha \right)+\left(1-\alpha \right)\log_{2}\left(m-1\right)$ numerically for all five channels using the grand-pooled transition matrices; Table 8 shows the actual H(Y|X), the bound, and the slack (bound minus actual) for each channel.

The slack quantifies how far the actual channel is from the worst-case (maximally diffuse off-diagonal structure) assumed by the bound. The bound is tightest for the pupil channel (slack =0.058 bits), where high persistence leaves little room for off-diagonal entropy, and for the direction channel (slack =0.072 bits), which has eight states and a relatively diffuse transition structure. The bound is loosest for AOI (slack =0.309 bits): despite moderate α, its rich spatial composition produces a highly structured off-diagonal pattern that keeps H(Y|X) well below the worst case, illustrating that α alone does not determine H(Y|X)—off-diagonal structure matters too (Remark 1). The five channels occupy well-separated regions along the α axis (Figure 13): direction (α≈0.23) and amplitude (α≈0.57) have the lowest persistence; AOI (α≈0.62) and duration (α≈0.65) have intermediate persistence; and the pupil channel (α≈0.86) has the highest persistence and the highest MI among the four non-AOI channels. This between-channel pattern is broadly consistent with the expectation that higher persistence is associated with greater sequential dependence, with AOI as the notable exception owing to its large state space. A noteworthy detail is that amplitude (α≈0.57, I≈0.029) and duration (α≈0.65, I≈0.046) are the two lowest-MI channels, both well below the direction, pupil, and AOI channels, despite representing different physical quantities—consistent with both being components of the ambient/focal scanning mode captured by Krejtz et al.’s κ coefficient [10]. Their cross-channel Spearman correlation is nonetheless not significant (participants: ρ=+0.418, p=0.229; paintings: ρ=−0.091, p=0.779), confirming that they carry similar *amounts* of sequential structure but not the *same* structure. Within each channel, α varies only modestly across paintings and participants, so the dominant source of MI variation within a channel is the shape of the off-diagonal transition structure rather than persistence alone.

The pupil diameter channel shows the second highest I(X;Y) of all five channels (after the AOI channel), which is consistent with the slow physiological dynamics of pupil dilation [14]. Pupil size reflects both psychosensory and luminance-driven processes, which unfold over seconds and are thus inherently persistent across consecutive fixations; this physiological timescale explains why self-transition probabilities are very high across both aggregations (around 0.78 for Small, 0.88 for Medium, and 0.90 for Large, nearly identical across participants and paintings) and why I(X;Y) substantially exceeds the amplitude, duration, and direction channels. The near-zero Pupil–Duration painting-level correlation (ρ=+0.028) stands in contrast to the nominally significant participant-level correlation (ρ=+0.697). The sensitivity analysis shows that this participant-level result is not robust, losing significance upon removal of P01 (ρ=+0.583, p=0.099).

The overall pattern of weak and statistically non-significant cross-channel associations suggests that the channels may capture partially distinct aspects of gaze behaviour, although the present sample size does not allow strong conclusions regarding channel independence.

A connection worth noting is that the amplitude and duration channels together correspond to the components of Krejtz et al.’s κ coefficient [10], which quantifies the temporal correlation between fixation duration and subsequent saccade amplitude as a marker of the ambient–focal distinction. Despite this conceptual link, the Spearman correlation between duration and amplitude I(X;Y) values is not significant at either level (participants: ρ=+0.418, p=0.229; paintings: ρ=−0.091, p=0.779), suggesting that the channel measures capture different aspects of these dimensions than the scalar κ statistic does.

The relationship between channel measures and computational aesthetics quantities [1] is examined in Section 4.11. Notably, the pupil channel shows a nominally significant negative correlation with Bense’s palette redundancy $M_{B}$ (ρ=−0.692, p=0.013, uncorrected), the AOI channel shows a marginal positive correlation with compositional complexity $M_{s}^{-1}$ (ρ=+0.503, p=0.095), and the direction channel shows a marginal positive correlation with $M_{k}$ (ρ=+0.510, p=0.090). By contrast, neither permutation entropy $H_{\mathrm{PE}}$ nor statistical complexity $C_{\mathrm{PE}}$ shows a significant association with any channel, likely because all 12 Van Gogh paintings occupy a compressed high-entropy range that limits between-painting discrimination.

## 6. Limitations

Several limitations of this study should be noted.

**Sample size.** The dataset comprises 10 observers and 12 paintings. The small *N* limits statistical power for cross-channel association tests (N=10 or N=12 per Spearman correlation) and means that individual outlier participants can substantially influence results, as shown by the sensitivity analysis for the Pupil–Duration pair. Replication with larger and more diverse samples is necessary before drawing strong conclusions.

**Single dataset and stimulus type.** All 12 stimuli are paintings by a single artist. The channel properties—particularly the finding that amplitude discriminates observers better than paintings while pupil discriminates observers better than paintings—may not generalise to other stimulus types (natural scenes, faces, diagrams) or tasks (visual search, reading).

**Luminance confound in the pupil channel.** As noted in Section 3.3, fixation-period pupil size reflects a mixture of psychosensory arousal and the pupil light response to local luminance [14]. Without per-fixation luminance estimates, these contributions cannot be separated. The pupil–$M_{B}$ correlation (ρ=−0.692) should therefore be interpreted with caution: it may partly reflect structured luminance transitions in paintings with a more ordered, limited palette (high $M_{B}$) rather than purely cognitive differences in arousal.

**Choice of channel input order.** The channel $X_{t}\to X_{t+1}$ constructed in this paper is a valid information channel regardless of whether the underlying gaze process is first-order Markov or not: the transition matrix P(Y|X) and the mutual information $I(X_{t};X_{t+1})$ are well-defined descriptors of the sequential structure between consecutive fixation states. The question of Markov order is therefore not one of validity but of completeness: a second-order channel with compound input $\left(X_{t-1},X_{t}\right)$ captures strictly more sequential dependence than the first-order channel, since by the data-processing inequality $I\left(X_{t};X_{t+1}\right)\leq I\left(X_{t-1},X_{t};X_{t+1}\right)$. Bonev et al. [7] reported empirical evidence of higher-order Markovian patterns in saccade length sequences (pixel displacement), suggesting that a second-order channel may capture additional sequential dependence beyond what is reported here. The standard tool for testing whether this additional structure is statistically detectable is the asymptotic likelihood-ratio G-test, but for the second-order amplitude channel (9×3 transition matrix, 27 cells) the present dataset is too sparse to satisfy the Cochran condition for the $\chi^{2}$ approximation in most units, so this question cannot be answered reliably here. A larger dataset would allow both reliable G-test evaluation and more stable estimation of the second-order channel MI.

**Discretisation choices and pupil threshold bias.** All channel measures depend on the choice of category boundaries. Although the amplitude channel showed robustness across two categorisation schemes (Remark 2 guarantees that finer discretisation cannot decrease MI), alternative binning strategies could alter absolute MI values and transition structures. The choice of boundaries for the pupil channel was motivated by the empirical distribution of the data; different datasets or viewing conditions may require different thresholds. More importantly, fixed absolute thresholds are a limitation for the pupil channel because individual baseline pupil diameter varies with age, iris pigmentation, and ambient illumination [14]. Although the present experiment used constant room illumination and a single recording session per observer, the absolute categories (Small/Medium/Large) may not represent equivalent physiological states for all participants. A more principled alternative would be to normalise each observer’s pupil diameters (e.g., by per-observer z-score or tertile split) before assigning categories, producing equal-frequency states per observer and eliminating inter-individual baseline differences. Adopting this participant-normalised scheme would not be expected to alter the qualitative pattern of results (pupil showing the highest MI among the two new channels) but would change the exact numerical values and potentially the cross-channel correlation results; this is noted as a direction for future work.

**Multiple comparisons.** The exploratory correlation analyses in Section 4.11 involve 25 pairwise tests across five channels and five aesthetic measures. With a nominal significance level of 0.05 (uncorrected), up to one or two false positives would be expected by chance alone. The single nominally significant result (Pupil–$M_{B}$, p=0.013) does not survive Bonferroni correction (threshold p<0.002) and should therefore be treated as hypothesis-generating rather than confirmatory.

## 7. Conclusions

This paper introduced two new gaze information channels: the saccade amplitude channel and the pupil diameter channel. Applied to 10 observers viewing 12 Van Gogh paintings:

- The amplitude channel shows that observer-driven variation exceeds stimulus-driven variation in this dataset, in contrast to the AOI channel.
- The pupil channel shows the highest I(X;Y) among the two new channels (participant mean 0.489 bits, painting mean 0.746 bits), which is consistent with the slow dynamics of pupil responses.
- Two theoretical observations (Proposition 1 and Remark 2) provide a simple upper bound on the conditional entropy in terms of transition persistence, and show that state-space refinement cannot decrease channel mutual information, directly explaining the 3-category to 4-category increase in amplitude MI.
- Goodness-of-fit tests confirm significantly non-random sequential structure in both channels at p<0.01 for all pooled matrices.
- Of the 20 pairwise Spearman correlations across five channels, 19 did not reach statistical significance, suggesting that the channels may capture partially distinct aspects of gaze behaviour, although the present sample size does not allow strong conclusions regarding channel independence; the single nominally significant result (Pupil–Duration, ρ=+0.697) does not survive Bonferroni correction and is not robust to removal of one outlier participant.
- In an exploratory comparison with five computational aesthetics measures, the pupil channel I(X;Y) shows a nominally significant negative correlation with Bense’s palette redundancy $M_{B}$ (ρ=−0.692, p=0.013, uncorrected, does not survive Bonferroni correction): paintings with more diverse colour palettes are associated with stronger sequential pupil dynamics, though this result is hypothesis-generating only, and the contribution of luminance-driven pupil responses cannot be separated from cognitive effects without per-fixation luminance data. The AOI channel shows a marginal positive association with compositional complexity $M_{s}^{-1}$ (ρ=+0.503, p=0.095), and the direction channel with Kolmogorov compressibility $M_{k}$ (ρ=+0.510, p=0.090). Permutation entropy $H_{\mathrm{PE}}$ and statistical complexity $C_{\mathrm{PE}}$ show no significant association with any channel, consistent with the narrow high-entropy range occupied by all 12 Van Gogh paintings.

Future work should pursue several directions. First, both channels should be validated on larger and more diverse datasets spanning different stimulus types (natural scenes, visual search, reading), to establish whether the observer-versus-stimulus discrimination pattern reported here generalises beyond art viewing. Second, the Pupil–Duration association should be tested with an independent measure of arousal or cognitive load (e.g., task-evoked pupillary response, skin conductance, or subjective workload rating) to determine whether the shared variance is genuinely cognitive or partly artifactual. Third, a luminance-corrected version of the pupil channel should be computed once per-fixation luminance values are available, to disentangle psychosensory and light-response contributions to the sequential structure. Fourth, extending the channel input from $X_{t}$ to the compound state $\left(X_{t-1},X_{t}\right)$ would define a second-order channel that, by the data-processing inequality, captures at least as much sequential dependence as reported here. Whether this additional structure is practically detectable requires a larger dataset: the second-order amplitude channel (9×3 transition matrix) is too sparse for reliable G-test evaluation in the present data [7]. Finally, additional channels—such as saccade velocity or blink rate — could further enrich the multi-channel framework.

## Figures and Tables

**Table 1:** Amplitude channel measures per participant (up panel) and per painting (bottom panel).

Participants					
		3-cat		4-cat	
Part.	*N*	H(X)	I(X;Y)	H(X)	I(X;Y)
P01	1811	1.212	0.014	1.580	0.031
P02	1367	1.034	0.020	1.465	0.021
P03	1159	1.151	0.021	1.518	0.032
P04	452	1.173	0.061	1.580	0.091
P05	1360	1.198	0.089	1.653	0.100
P06	1310	1.155	0.009	1.535	0.015
P07	1273	1.094	0.011	1.532	0.028
P08	1123	1.080	0.035	1.423	0.039
P09	1143	1.080	0.009	1.393	0.014
P10	1179	1.090	0.018	1.319	0.027
Mean		1.127	0.029	1.500	0.040
SD		0.059	0.026	0.100	0.031
Paintings					
		3-cat		4-cat	
Paint.	*N*	H(X)	I(X;Y)	H(X)	I(X;Y)
a1	921	1.158	0.019	1.602	0.032
a2	516	1.157	0.051	1.571	0.067
a3	1094	1.113	0.050	1.455	0.052
a4	1051	1.169	0.032	1.533	0.040
a5	958	1.102	0.039	1.353	0.068
a6	1100	1.114	0.025	1.337	0.031
b1	749	1.058	0.036	1.427	0.047
b2	1255	1.153	0.010	1.523	0.018
b3	982	1.164	0.010	1.530	0.017
b4	1114	1.153	0.012	1.542	0.027
b5	1302	1.134	0.011	1.625	0.019
b6	1135	1.064	0.022	1.519	0.025
Mean		1.128	0.026	1.501	0.037
SD		0.038	0.015	0.091	0.018

**Table 2:** I(X;Y) (bits) for all five channels per participant (up panel) and per painting (bottom panel). AOI values from Hao et al. [1]. SD: sample standard deviation (N−1 denominator).

Participants					
Part.	AOI	Amp	Dur	Dir	Pup
P01	1.244	0.014	0.000	0.047	0.144
P02	1.253	0.020	0.071	0.161	0.617
P03	1.351	0.021	0.021	0.286	0.423
P04	1.205	0.061	0.075	0.230	0.771
P05	1.364	0.089	0.031	0.173	0.732
P06	1.309	0.009	0.046	0.217	0.694
P07	1.216	0.011	0.024	0.238	0.300
P08	1.399	0.035	0.117	0.140	0.459
P09	1.444	0.009	0.041	0.237	0.301
P10	1.456	0.018	0.035	0.164	0.448
Mean	1.324	0.029	0.046	0.189	0.489
SD	0.093	0.026	0.034	0.067	0.209
Paintings					
Paint.	AOI	Amp	Dur	Dir	Pup
a1	1.150	0.019	0.080	0.205	0.480
a2	1.193	0.051	0.112	0.222	0.747
a3	1.334	0.050	0.081	0.130	0.927
a4	1.359	0.032	0.038	0.209	0.795
a5	1.530	0.039	0.051	0.148	0.600
a6	1.680	0.025	0.063	0.195	0.624
b1	1.142	0.036	0.102	0.274	0.995
b2	1.524	0.010	0.077	0.151	0.545
b3	1.280	0.010	0.063	0.116	0.827
b4	1.304	0.012	0.104	0.062	0.708
b5	1.517	0.011	0.071	0.115	0.928
b6	1.391	0.022	0.132	0.105	0.773
Mean	1.367	0.026	0.081	0.161	0.746
SD	0.169	0.015	0.027	0.060	0.161

**Table 3:** Observer-level vs. stimulus-level discrimination by channel, measured as the sample standard deviation of I(X;Y) across participants ($\sigma_{\mathrm{obs}}$, N=10) and across paintings ($\sigma_{\mathrm{stim}}$, N=12). A ratio >1 indicates better discrimination of paintings than observers.

Channel	$\sigma_{\mathrm{obs}}$	$\sigma_{\mathrm{stim}}$	Ratio	Discriminates
AOI	0.093	0.169	1.82	stimuli
Amplitude	0.026	0.015	0.58	observers
Duration	0.034	0.027	0.79	observers
Direction	0.067	0.060	0.90	observers
Pupil	0.209	0.161	0.77	observers

**Table 4:** Pupil diameter channel H(X) and I(X;Y) per participant (up panel) and per painting (bottom panel).

Participants			
Part.	*N*	H(X)	I(X;Y)
P01	1811	0.533	0.144
P02	1367	1.109	0.617
P03	1159	1.221	0.423
P04	452	1.322	0.771
P05	1360	1.342	0.732
P06	1310	1.492	0.694
P07	1273	0.811	0.300
P08	1123	1.046	0.459
P09	1143	0.843	0.301
P10	1179	0.959	0.448
Mean		1.068	0.489
SD		0.291	0.209
Paintings			
Painting	*N*	H(X)	I(X;Y)
a1	921	0.903	0.480
a2	516	1.218	0.747
a3	1094	1.522	0.927
a4	1051	1.530	0.795
a5	958	1.297	0.600
a6	1100	1.290	0.624
b1	749	1.304	0.995
b2	1255	1.270	0.545
b3	982	1.555	0.827
b4	1114	1.403	0.708
b5	1302	1.412	0.928
b6	1135	1.284	0.773
Mean		1.332	0.746
SD		0.177	0.161

**Table 5:** Spearman ρ across participants (N=10, up panel) and paintings (N=12, bottom panel). * p<0.05 uncorrected; does not survive Bonferroni correction.

Participants (N=10)					
	AOI	Amp	Dur	Dir	Pup
AOI	—	+0.03	+0.08	−0.12	−0.18
Amp		—	+0.42	−0.41	+0.36
Dur			—	−0.21	+0.70 *
Dir				—	+0.02
Pup					—
Paintings (N=12) — no entry significant					
	AOI	Amp	Dur	Dir	Pup
AOI	—	−0.18	−0.50	−0.34	−0.16
Amp		—	−0.09	+0.00	+0.38
Dur			—	−0.15	+0.03
Dir				—	−0.34
Pup					—

**Table 6:** Spearman ρ between per-painting I(X;Y) and aesthetics measures. $M_{k}$: Kolmogorov compressibility [1,17]; $M_{B}$: Bense palette redundancy [1,17]; $M_{s}^{-1}$: compositional complexity (number of regions capturing 25% of colour–space MI) [17]; $H_{\mathrm{PE}}$: normalised permutation entropy [18]; $C_{\mathrm{PE}}$: statistical complexity [18]. $H_{\mathrm{PE}}$ and $C_{\mathrm{PE}}$ were computed from the painting images following the Sigaki et al. method (2 × 2 sliding window, 24 ordinal patterns). * p<0.05; † p<0.10.

Channel	$\rho (M_{k})$	*p*	$\rho (M_{B})$	*p*	$\rho (M_{s}^{-1})$	*p*	$\rho (H_{\mathrm{PE}})$	*p*	$\rho (C_{\mathrm{PE}})$	*p*
AOI	−0.21	0.513	−0.01	0.966	${+0.50}^{\;†}$	0.095	+0.00	1.000	+0.00	1.000
Amplitude	−0.06	0.846	−0.41	0.182	+0.25	0.429	+0.01	0.974	−0.01	0.974
Duration	−0.12	0.712	+0.16	0.609	−0.08	0.803	+0.48	0.117	−0.48	0.117
Direction	${+0.51}^{\;†}$	0.090	+0.20	0.542	−0.41	0.191	−0.29	0.366	+0.29	0.366
Pupil	−0.48	0.118	−0.69 *	0.013	+0.29	0.366	+0.07	0.829	−0.07	0.829

**Table 7:** Per-painting values of $H_{\mathrm{PE}}$ (normalised permutation entropy) and $C_{\mathrm{PE}}$ (statistical complexity) corresponding to the aesthetics measures used in Table 6. Computed from painting images following Sigaki et al. [18] (2 × 2 sliding window, 24 ordinal patterns).

Painting	$H_{\mathrm{PE}}$	$C_{\mathrm{PE}}$	Period	Painting	$H_{\mathrm{PE}}$	$C_{\mathrm{PE}}$	Period
a1	0.969	0.040	1	b1	0.983	0.023	1
a2	0.979	0.027	2	b2	0.957	0.056	2
a3	0.961	0.049	3	b3	0.934	0.085	3
a4	0.944	0.070	4	b4	0.995	0.007	4
a5	0.991	0.012	5	b5	0.980	0.026	5
a6	0.968	0.041	6	b6	0.989	0.014	6

**Table 8:** Numerical verification of Proposition 1. H(Y|X): actual conditional entropy; bound: $H_{b}\left(\alpha \right)+\left(1-\alpha \right)\log_{2}\left(m-1\right)$; slack: bound − actual (must be ≥0).

Channel	*m*	α	H(Y|X)	Bound	Slack
AOI	10	0.618	1.860	2.169	0.309
Amplitude	3	0.572	1.121	1.413	0.292
Duration	3	0.649	1.035	1.287	0.252
Direction	8	0.228	2.870	2.942	0.072
Pupil	3	0.860	0.668	0.726	0.058

## Data Availability

The eye-tracking dataset used in this study is available from the corresponding authors upon reasonable request.

## Abbreviations

AOI	Area of Interest
MI	Mutual Information
SD	Short Duration
MD	Medium Duration
LD	Long Duration
